# Oleic Acid-Shelled Solid Lipid Nanocomplexes for Enhanced Quercetin Delivery with Improved Antioxidant and Stability Properties

**DOI:** 10.1021/acsomega.5c04754

**Published:** 2025-07-16

**Authors:** Xuwei Shang, Fuqiang Hu

**Affiliations:** College of Pharmaceutical Science, 12377 Zhejiang University, 866 Yuhangtang Road, Hangzhou 310058, People’s Republic of China

## Abstract

Quercetin (Que), a bioactive flavonoid with anti-inflammatory and antioxidant effects, faces limited use due to its poor solubility and instability. To overcome these limitations, a novel glyceryl monostearate (GM)-based solid lipid nanocomplex was developed, incorporating oleic acid (OA) to improve stability and Que delivery. GM, aided by positively charged octadecylamine, encapsulated Que (forming LNP@Que), which was then electrostatically coated with OA to yield LNP@Que/OA. Optimizing the GM-to-OA mass ratio to 9:1 yielded a nanocomplex with a particle size of 183.3 ± 20.6 nm and a reduced zeta potential. The OA modification significantly improved the antioxidant properties, degradation resistance, and overall stability of the Que nanocomplex. Notably, LNP@Que/OA (10% OA + 90% GM) demonstrated the highest encapsulation efficiency (97.49%) and enabled effective controlled release of Que during in vitro simulated digestion. This study highlights the potential of LNP@Que/OA for pharmaceutical applications.

## Graphical abstract

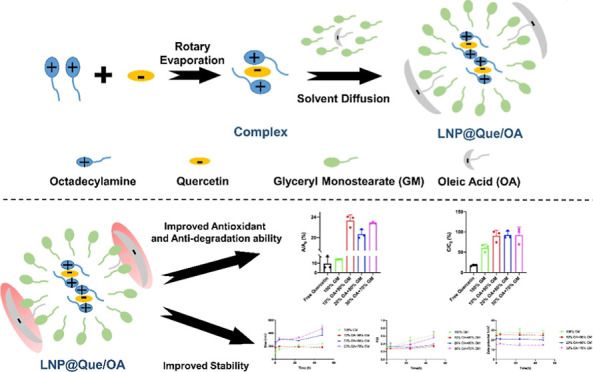

## 1 Introduction

Quercetin (Que) is a natural plant-based flavonoid found abundantly in many foods, including fruits, vegetables, and grains.^1^ Known for its antioxidant and anti-inflammatory properties, Que offers numerous health benefits.^2^ Due to its strong antioxidant activity, it could also serve as an effective natural preservative, helping to prevent oxidative damage in food products—reducing rancidity and maintaining quality. Specifically, quercetin (Que) can be utilized in oils, fats, and processed foods to extend shelf life,^3^ as well as incorporated into functional beverages and snacks to improve stability. Beyond its antioxidant function, Que can also serve as a health-enhancing ingredient in functional foods and beverages targeting health-conscious consumers.^4,5^ However, several challenges must be overcome before its widespread application. First, Que’s poor solubility limits its practical use.^6^ Additionally, as a polyphenolic compound with multiple hydroxyl (−OH) groups, Que is highly reactive under certain conditions, leading to potential degradation.^5^ Its light sensitivity further complicates storage, as exposure to sunlight or artificial light can trigger photo-oxidation, breaking it down into inactive byproducts.^7^ Moreover, factors such as high temperature, pH fluctuations, and oxygen exposure can significantly impact Que’s stability. Therefore, developing effective strategies to enhance Que’s solubility and protect it from degradation is critically needed.

Nanomedicine-based drug delivery systems have emerged as a transformative approach in modern therapeutics, offering unprecedented opportunities to enhance drug solubility, improve targeted delivery, improve stability, and reduce systemic toxicity. By leveraging the unique physicochemical properties of nanoparticles (e.g., liposomes, polymeric NPs, and inorganic carriers), these systems enable precise control over drug release kinetics and tissue-specific accumulation.

Solid lipid nanoparticles (LNPs), composed of physiologically biocompatible lipids, have demonstrated significant potential in drug delivery applications.^8^ These nanoparticles effectively enhance drug solubility, improve cellular uptake, increase stability, reduce enzymatic degradation, and prolong systemic circulation time for various therapeutic compounds.^9^ Notably, LNPs exhibit superior encapsulation efficiency for both hydrophilic and hydrophobic drugs compared to traditional liposomes.^10^ The versatility of LNPs is evidenced by their successful application across multiple administration routes, including oral,^11^ parenteral,^12^ transdermal,^13^ intranasal,^14^ ocular,^15^ and pulmonary^16^ delivery systems. These applications consistently show improved drug safety profiles, enhanced bioavailability, and optimized therapeutic outcomes. For Que delivery, LNPs present a particularly promising solution by potentially overcoming the compound’s inherent solubility limitations and stability challenges while enabling targeted delivery. This makes them excellent candidates for pharmaceutical applications. However, several technical challenges currently limit the widespread adoption of the LNP technology. Stability concerns remain paramount. LNPs may undergo physical instability (e.g., Ostwald ripening or gelation) during storage, leading to an increased particle size and reduced efficacy. The crystalline lipid matrix can undergo polymorphic transitions, causing premature drug expulsion (especially for hydrophilic or small-molecule drugs like quercetin). Addressing these stability issues represents a critical research priority for advancing LNP-based delivery systems.

Oleic acid (OA), a monounsaturated omega-9 fatty acid naturally present in both animal and plant-derived fats and oils,^17^ exists as a colorless and odorless oil in pure form but often appears yellowish in commercial grades due to impurities. This versatile compound serves multiple pharmaceutical functions as an excipient, particularly as an effective emulsifier and solubilizing agent in aerosol formulations. Beyond its formulation applications, studies found that OA can facilitate skin wound healing and would be a potential induction factor for tissue engineering.^18^ In addition, OA demonstrates significant biochemical activity by modulating antioxidant enzyme synthesis and function while exhibiting exceptional oxidative stability that can potentiate the efficacy of coformulated antioxidants.^19^ When applied as a surface coating for LNPs, OA confers enhanced stability to the nanoparticle system while providing protective effects against Que oxidation, making it particularly valuable for optimizing LNP-based delivery systems for sensitive compounds.

In this study, we developed a Que delivery system by first encapsulating Que in glyceryl monostearate (GM) with positively charged octadecylamine to form LNP@Que, then coating it with negatively charged OA via electrostatic interactions to produce LNP@Que/OA. To evaluate its pharmaceutical potential, we systematically investigated how GM-to-OA ratios affect performance through three key analyses: (1) physicochemical characterization including morphology, encapsulation efficiency, and FT-IR spectroscopy; (2) evaluation of Que release kinetics, storage stability, and ratio optimization; and (3) assessment of antioxidant capacity, degradation resistance, and biosafety. This comprehensive approach enables the rational design of LNP@Que/OA systems tailored for pharmaceutical applications, with the GM-to-OA ratio serving as a critical adjustable parameter to optimize stability, release properties, and functional performance.

## 2 Materials and Methods

### 2.1 Materials

Octadecylamine, GM, d-mannitol, potassium ferricyanide, and quercetin were purchased from Macklin. OA, iron­(III) chloride hexahydrate, and trichloroacetic acid were obtained from Aladdin. Ethanol and tween-80 were purchased from Sinopharm Chemical Reagent Co., Ltd., China.

### 2.2 Preparation of LNP@Que/OA NPs

20 mg of quercetin and 40 mg of octadecylamine were weighed, dissolved in 8 mL of ethanol, and rotary evaporation was performed at 60 °C and 90 rpm for 15 min to obtain the complex. It was redissolved in 16 mL of ethanol, and a mass fraction of OA and GM (100% GM; 10% OA + 90% GM; 20% OA + 80% GM; 30% OA + 70% GM) was added 9 times that of Que, dissolved at 60 °C and 400 rpm, and then added to 160 mL of purified water. It was stirred for 10 min, then cooled to room temperature. Then, it was centrifuged at 8000 rpm for 10 min at 25 °C and the supernatant was collected. Finally, mannitol (1%, w/v) was added and freeze-dried for 2 days to obtain the LNP@Que/OA powder.

### 2.3 Characterization of LNP@Que/OA NPs

The particle size distribution and polydispersity (PDI) of LNP@Que/OA NPs were determined by a Nano-ZS90 Malvern Mastersizer (Malvern Instruments Ltd., Malvern, UK) at 25 °C. The zeta potential of LNP@Que/OA NPs was determined by a ZETASIZER LAB Malvern (Malvern Instruments Ltd., Malvern, UK) at 25 °C. The morphology of LNP@Que/OA NPs was analyzed with a JEM-1400 transmission electron microscope (TEM) (JEOL, Japan). First, the TEM grid was prepared by glow-discharging a carbon-coated copper grid for 30–60 s to enhance hydrophilicity and improve LNP adhesion. The LNP suspension was diluted in ultrapure water to a ratio of 1:10–1:100 to prevent aggregation. 3–5 μL of the diluted sample was pipetted onto the grid and allowed it to adsorb for 1–2 min. For contrast enhancement, 3–5 μL of uranyl acetate (a standard negative stain for lipids) was applied and incubated for 30 s. Excess liquid was gently blotted using filter paper, ensuring that the grid surface remains intact. The grid was air-dried for 10–15 min in a clean, dust-free environment before TEM insertion. The FT-IR spectrum of LNP@Que/OA NPs was monitored by a NICOLET iS50FT-IR spectrometer (Thermo Scientific, US). 10 mg of Que was weighed and dissolved in 50 mL of ethanol to prepare a 200 μg/mL mother liquor. It was diluted with an appropriate amount of ethanol to achieve a series of Que concentrations of 1, 5, 10, 20, and 40 μg/mL. The absorbance values of Que-ethanol solution were determined by UV–vis spectroscopy by measurement at the absorption wavelength of 378 nm. Linear regression was performed on absorbance values using Que concentration (μg/mL), and the standard curve was plotted. According to the standard curve, the amount of Que encapsulated in the nanoparticles was determined by UV–vis spectroscopy by measurement at an absorption wavelength of 378 nm. The Que encapsulation efficiency (EE %) and drug loading (DL %) values were calculated by the following equations

EE%=weightofQueinNPs/weightofQuefed×100%

DL%=weightofQueinNPs/weightofNPs×100%

### 2.4 Stability of LNP@Que/OA NPs

The storage temperature stabilities of LNP@Que/OA NPs were carried out at room temperature, respectively. At the proper times (0, 4, 24, and 48 h), the size distribution and PDI of LNP@Que/OA nanoparticles were monitored by a nano-ZS90 Malvern Mastersizer (Malvern Instruments Ltd., Malvern, UK) at 25 °C. In addition, the zeta potential of LNP@Que/OA nanoparticles was determined by a ZETASIZER LAB Malvern (Malvern Instruments Ltd., Malvern, UK) at 25 °C. All samples were measured in triplicate.

### 2.5 In Vitro Release Assay

Dialysis was used to determine the in vitro release profiles. LNP@Que/OA NPs were suspended in PBS (pH 6.8 or pH 7.4). A suspension with an equivalent concentration of Que (0.25 mg mL^–1^, 2 mL) was dialyzed against 30 mL of buffer (pH6.8 PBS or pH7.4 PBS with 0.5% tween-80) (MWCO = 3500 Da) in an incubator shaker at 37 °C with stirring at 60 rpm. At predetermined time intervals, 2 mL of release medium was removed and the same volume of fresh release medium was added to the dialysis tube. The samples were filtered through a 0.22 μm filter, and their absorbance was measured by UV–vis spectroscopy at 378 nm.

### 2.6 Antioxidant Activity

An appropriate amount of the formulation (Que; 100% GM; 10% OA + 90% GM; 20% OA + 80% GM; 30% OA + 70% GM) was weighed and dissolved in an appropriate amount of purified water to achieve a quercetin content of 10 μg. At 50 °C, the above formulation was taken and incubated with 1.25 times the volume of 0.1% potassium ferricyanide solution +1.25 times the volume of pH 6.8 PBS for 20 min. After incubation, the mixture was cooled in an ice–water bath to room temperature and then added to 1.25 times the volume of 10% trichloroacetic acid solution and allowed to stand for 5 min. It was centrifuged at 3000 rpm for 10 min at 25 °C and the supernatant was collected. Next, 2 mL of the supernatant was taken, 2 mL of purified water and 0.4 mL of 0.1% ferric chloride were added and incubated at room temperature for 10 min. The absorbance (Abs) of each group was measured using UV spectroscopy with a maximum absorption wavelength of 700 nm. The Abs at Day 0 and after 7 days at room temperature was recorded as *A*_0_ and *A*, respectively, and the *A*/*A*_0_ ratio was calculated.

### 2.7 Antidegradation Activity

To explore the antidegradative activity, Que and LNP@Que/OA NPs suspensions (Que = 10 μg) were irradiated by a visible lamp for 6 days, and then the concentration of remnant Que was measured by an UV–vis spectrophotometer at 378 nm. Finally, one ratio, final concentration of Que/initial concentration of Que (*C*/*C*_0_), was calculated to study the degradation level of Que.

### 2.8 Hemolysis

An appropriate amount of LNP@Que/OA powder (10% OA + 90% GM; No mannitol) was weighed and dissolved in an appropriate amount of saline to achieve a series of carrier concentrations of 0.05, 0.1, 0.2, 0.3, 0.4, 0.6, 1.0, and 1.2 mg/mL. Saline and deionized water were selected as negative and positive controls, respectively. The same volume of newly prepared red blood cell dispersion (2%, v/v) was added, respectively. Red blood cells were first obtained from ICR mice. All animal experiments were approved by the Institutional Animal Care and Use Committee (IACUC) of Zhejiang University, School of Medicine, and the National Guidelines for Animal Protection was followed. It was incubated at 37 °C for 1 h. Then, it was centrifuged at 3000 rpm for 10 min at 4 °C and the supernatant was collected. The absorbance (Abs) of each group was measured using UV spectroscopy with a maximum absorption wavelength of 545 nm. The hemolysis % was calculated by the following equations

$$hemolysis\;\%=\left({Abs}_{simple}{-Abs}_{negative}{)/(Abs}_{positive}{-Abs}_{negative}\right)\times 100\%$$

### 2.9 Animals

ICR mice (6 weeks old, weight 20–24 g) were purchased from Shanghai SLAC Laboratory Animal Co., Ltd. Animals were kept at 20–25 °C under a 12 h light–dark cycle and obtained food and water freely. All studies were approved by the Zhejiang University Laboratory Animal Welfare Ethics Review Committee and were conducted in accordance with national regulations and protocols.

### 2.10 In Vivo Safety Assessment

All mice were randomly divided into 6 groups (3 mice per group). Then, the mice were orally administered (i) normal saline, (ii) quercetin, (iii) LNP@Que NPs (100% GM), (iv) LNP@Que/OA NPs (10% OA + 90% GM), (v) LNP@Que/OA NPs (20% OA + 80% GM), and (vi) LNP@Que/OA NPs (30% OA + 70% GM) every 2 days 5 times. The Que dosage for each group was 12.5 mg/kg. During the assay, the mortality, food habit, appearance change, abnormal behavior, and adverse signs of toxicity were observed every day. At 12th day past the administration, the main organs (heart, liver, spleen, lung, and kidney) of mice were collected and fixed with 4% paraformaldehyde. The hematoxylin and eosin (H&E) staining and serological biochemical analysis were also performed in this study to learn the in vivo safety.

### 2.11 Statistical Analysis

All quantitative data were presented as mean ± standard deviation. The statistical comparisons between two groups were assessed by an unpaired student *t*-test. The comparisons between multiple groups were analyzed by one/two-way ANOVA. *P* < 0.05 indicated that the difference was statistically significant, and *p* < 0.01 was considered highly significant.

## 3 Results

### 3.1 Preparation and Characterization of LNP@Que/OA NPs

In this investigation, we successfully synthesized LNP@Que/OA nanoparticles, as shown in Figure 1A. We further characterized their physical properties, including particle size distribution and zeta-potential values, as displayed in Figure 1B,C. After the coverage of OA, the particle sizes of LNP@Que/OA NPs increased, which were measured to be 106.1 ± 18.6 nm (100% GM), 183.3 ± 20.6 nm (10% OA + 90% GM), 233.7 ± 23.1 nm (20% OA + 80% GM), and 257.7 ± 17.8 nm (30% OA + 70% GM), respectively. The PDI were measured to be 0.305 ± 0.030 (100% GM), 0.276 ± 0.009 (10% OA + 90% GM), 0.335 ± 0.049 (20% OA + 80% GM), and 0.292 ± 0.010 (30% OA + 70% GM), respectively. Meanwhile, the zeta potentials decreased with the coverage of negatively charged OA, which were 23.4 ± 2.2 mV (100% GM), 22.8 ± 1.6 mV (10% OA + 90% GM), 20.2 ± 1.3 mV (20% OA + 80% GM), and 12.7 ± 0.6 mV (30% OA + 70% GM), respectively. It was found that particle size and PDI of nanoparticles that have been lyophilized (Figure 1E) and reconstituted showed negligible changes compared to freshly prepared nanoparticles, as displayed in Figure 1D.

**Figure 1:**
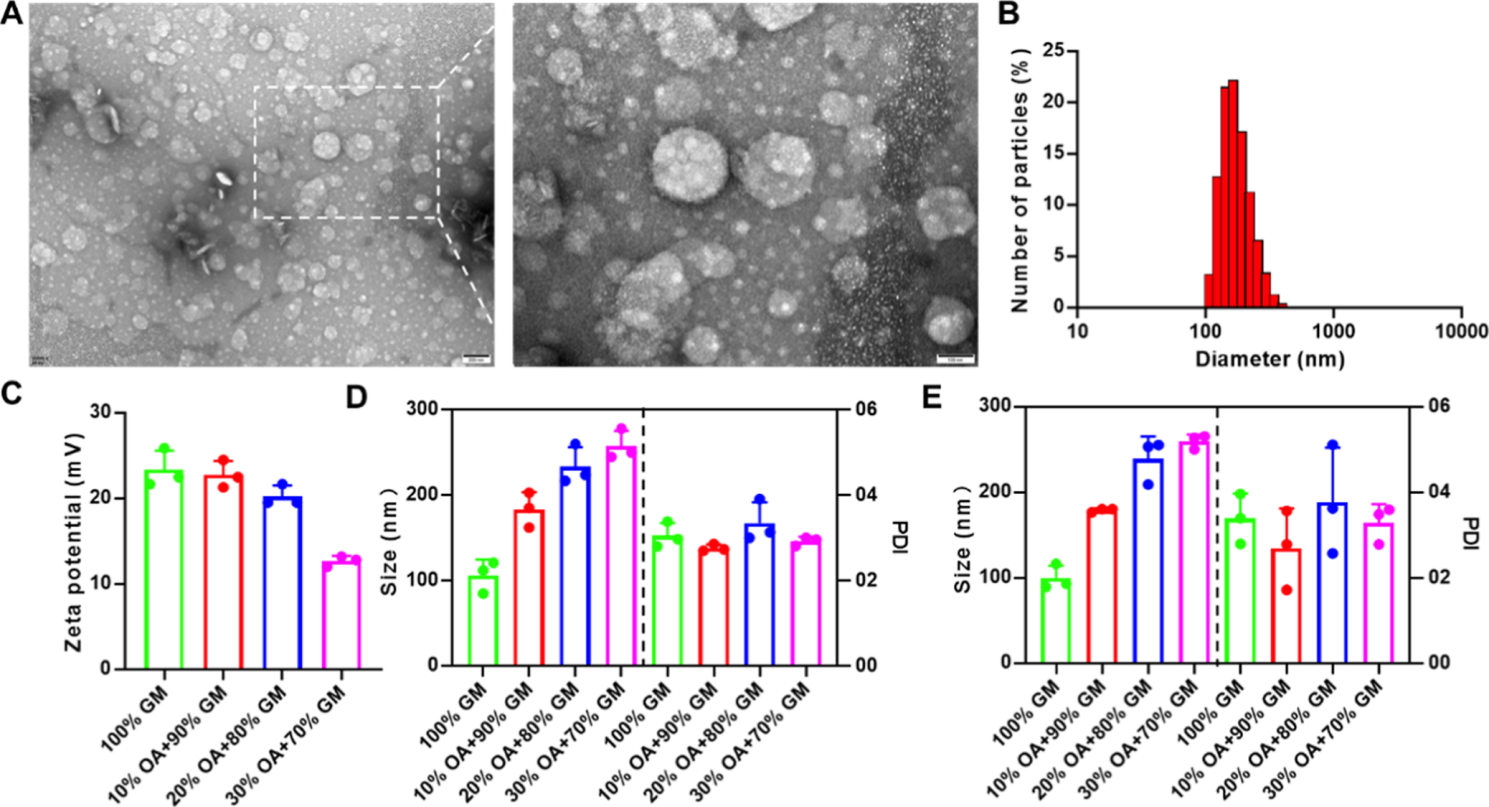
(A) TEM results, (B) particle distribution, and (C) zeta potential of LNP@Que/OA NPs, *n* = 3. Particle size and PDI of (D) newly prepared and (E) lyophilized LNP@Que/OA NPs, *n* = 3.

FT-IR spectroscopy was performed to characterize the chemical composition of the final LNP@Que/OA nanoparticles. As shown in Figure 2, the peak at 1736 cm^–1^ was attributed to the stretching vibration of –C=C– and –C=O in OA, octadecylamine, and GM. Meanwhile, the peaks in 1468 cm^–1^ and 2956/2918/2850 cm^–1^ were bending vibration and stretching vibration of –CH_2_–, CH_3_ in OA, octadecylamine, and GM, respectively. The broad peak at 3386 cm^–1^ belonged to the stretching vibration –OH and –NH_2_ in OA, octadecylamine, GM, and Que. The stretching vibration of –C=C– in the benzene ring of Que could be observed at 1561/1416 cm^–1^, indicating the successful loading of Que. Peaks at 1052 cm^–1^ and 1311/1266/1180 cm^–1^ were attributed to the bending vibration and stretching vibration of –C–O–C– in Que and the GM.

**Figure 2:**
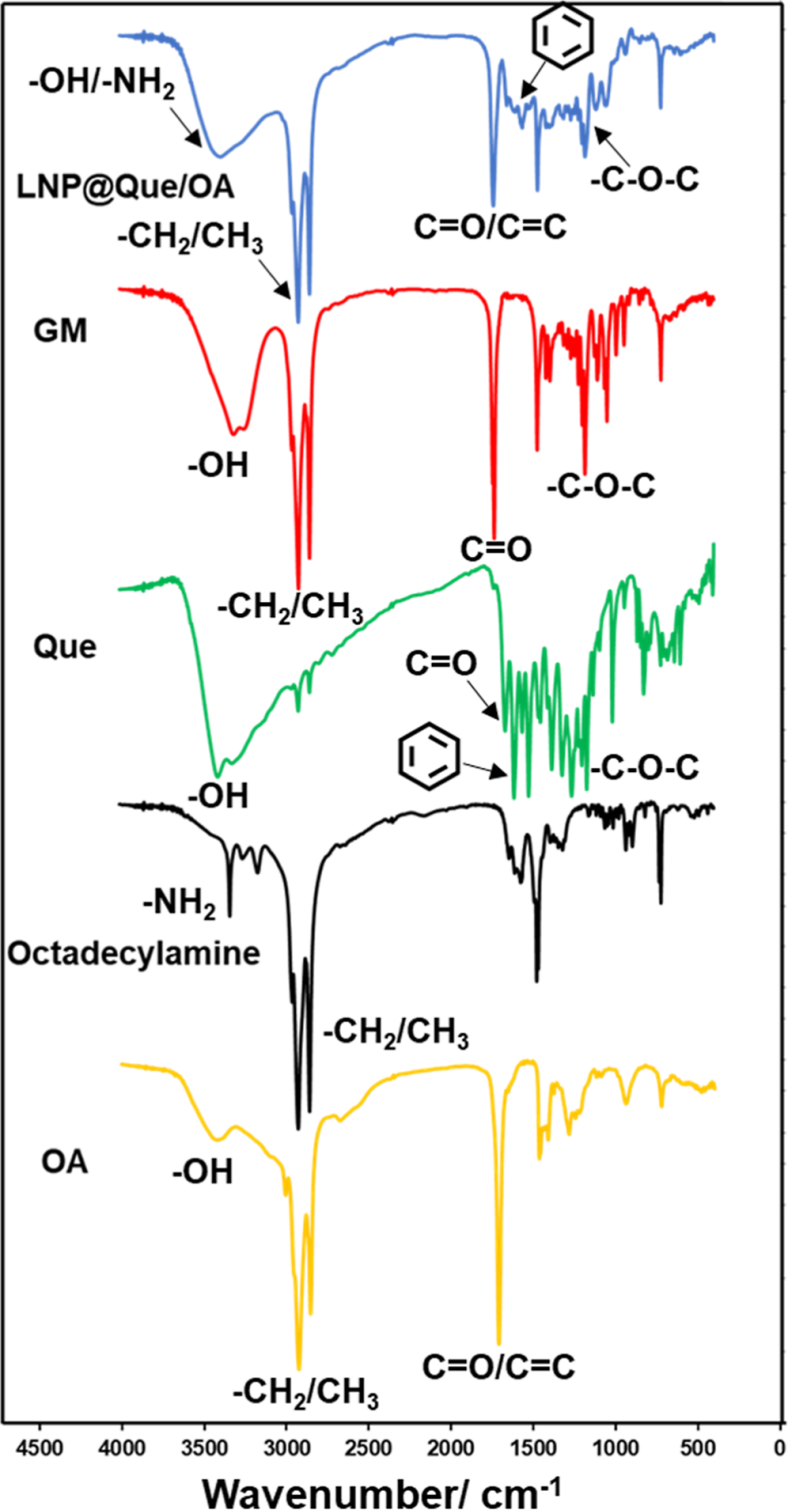
FT-IR results of Que, OA, GM, and LNP@Que/OA NPs.

### 3.2 In Vitro Release Evaluation

We determined the Que encapsulation efficiency (EE %) in LNP@Que/OA nanoparticles with varying GM-to-OA ratios by using UV–vis spectroscopy. Initial characterization of individual components (Que, OA, GM, mannitol, and octadecylamine) revealed that Que exhibits a distinct absorption peak at 378 nm (Figure 3A), absent in other formulation components. This characteristic peak enabled specific quantification of Que content, allowing precise measurement of EE % through absorbance readings at 378 nm. The standard curve is displayed in Figure 3B, which showed good linearity when the concentration of Que was 1 to 40 μg mL^–1^. The Que content in LNP@Que/OA NPs with different OA ratios was detected and is displayed in Figure 3C. LNP@Que/OA NPs (10% OA + 90% GM) had the highest Que encapsulation efficiency, up to 97.49%. The in vitro release behavior of LNP@Que/OA NPs was monitored by UV–vis. The DL % results were similar to that of EE %, as displayed in Figure 3C. As displayed in Figure 3D, the drug release from LNP@Que NPs (100% GM) at pH 6.8 rapidly increased to approximately 91.65% within 48 h, which was much slower than free Que, which reached 100% in 12 h. The drug release speed was ulteriorly restricted after the coverage of OA, which was detected to be around 84.30–84.93% within 48 h, which prevented the premature leakage of Que and promise the long-term release of Que in targeted places. The release profile at physiological pH (7.4) (Figure 3E) was similar to that at pH 6.8, although the release rate was slightly faster at pH 7.4. Kinetic studies revealed that the Higuchi models best fit the release data, as shown in Table 1.

**Figure 3:**
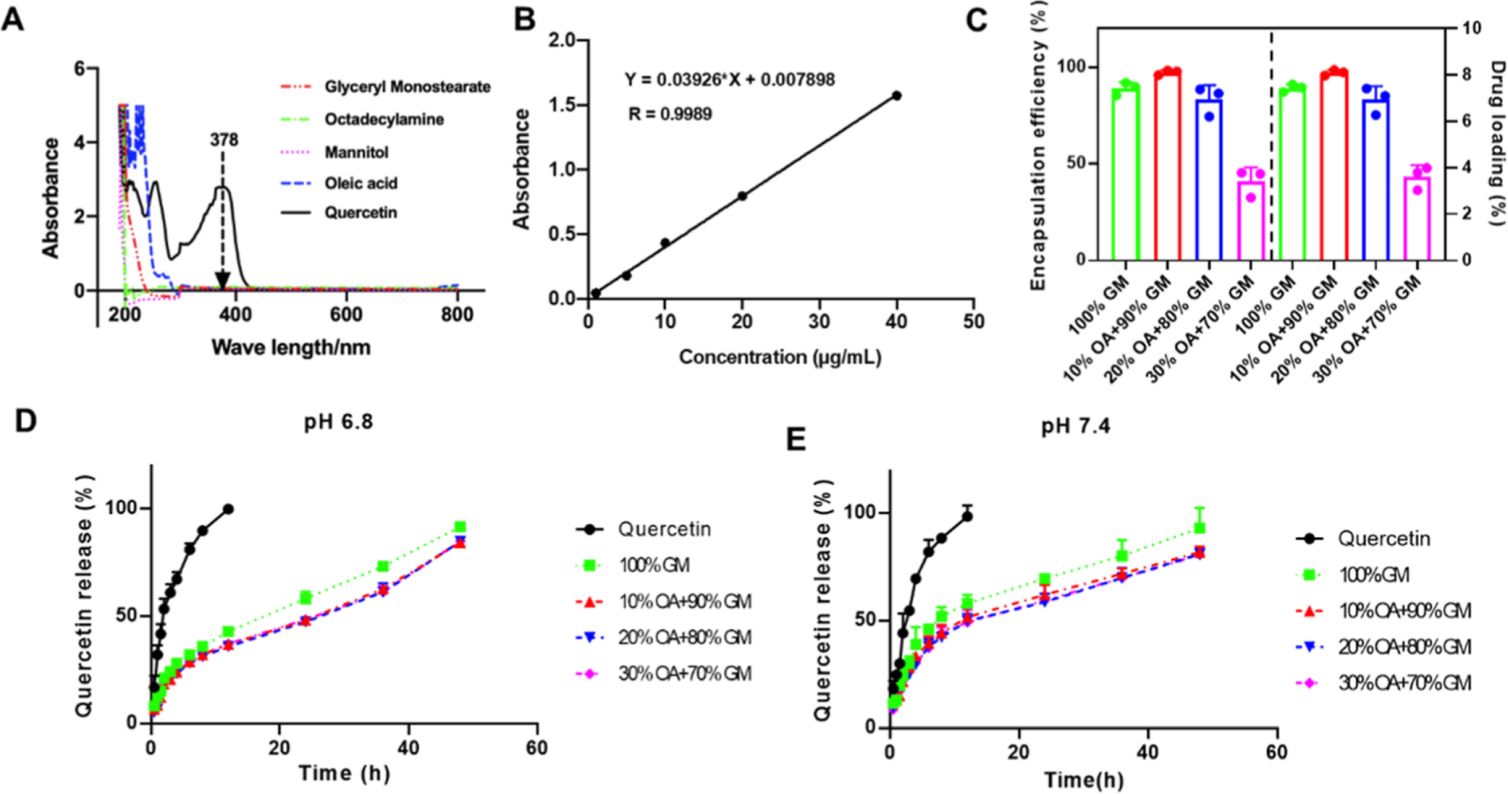
(A) UV–vis spectrum was measured to study the entrapment of Que and the coverage of OA by the final preparation. The (B) standard curve of Que measured by UV–vis. (C) Que encapsulation efficiency and drug loading of LNP@Que/OA NPs with different OA ratios. In vitro release behavior of LNP@Que/OA NPs in pH6.8 (D) and pH 7.4 (E), *n* = 3.

**Table 1:** Kinetic Studies and Analysis

group	Higuchi equation
100% GM-pH6.8	*Q* = 12.50 × *t* ^1/2^ + 0.8361, *R* ^2^ = 0.9928
10% OA + 90% GM-pH6.8	*Q* = 11.21 × *t* ^1/2^ – 0.3391, *R* ^2^ = 0.9805
20% OA +80% GM-pH6.8	*Q* = 11.20 × *t* ^1/2^ – 0.7639, *R* ^2^ = 0.9737
30% OA + 70% GM-pH6.8	*Q* = 10.98 × *t* ^1/2^ + 0.8991, *R* ^2^ = 0.9761
100% GM-pH7.4	*Q* = 13.19 × *t* ^1/2^ + 6.178, *R* ^2^ = 0.9632
10% OA + 90% GM-pH7.4	*Q* = 11.66 × *t* ^1/2^ + 5.443, *R* ^2^ = 0.9692
20% OA + 80% GM-pH7.4	*Q* = 11.45 × *t* ^1/2^ + 4.548, *R* ^2^ = 0.9761
30% OA + 70% GM-pH7.4	*Q* = 11.36 × *t* ^1/2^ + 5.233, *R* ^2^ = 0.9747

### 3.3 Stability of LNP@Que/OA NPs

We evaluated the stability of LNP@Que/OA nanoparticles by monitoring changes of particle size, PDI, and zeta potential over 48 h. The results in Figure 4A revealed distinct stability patterns among formulations with different OA percentages: formulations containing 0%, 20%, and 30% OA exhibited significant particle size increases over time, while the 10% OA formulation demonstrated excellent stability with negligible size variation. Meanwhile, the PDI almost did not change with 10% OA formulation (Figure 4B). Zeta potential did not show obvious changes in all formulation (Figure 4C). These findings indicate that the 10% OA formulation possesses optimal in vitro stability characteristics.

**Figure 4:**
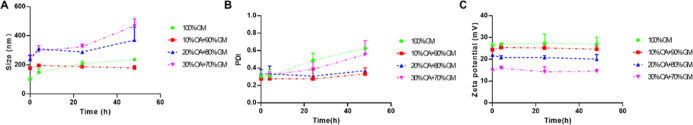
(A) Particle size, (B) PDI, and (C) zeta potential stability test, *n* = 3.

### 3.4 Protection of LNP@Que/OA NPs for Que

#### 3.4.1 Antidegradation Activity

Previous studies have demonstrated that Que undergoes rapid photochemical degradation when exposed to UV or visible light, regardless of whether it is in solution or solid form. To address this stability challenge, we evaluated the protective effects of OA modification on LNP@Que/OA nanoparticles. As shown in Figure 5A, OA surface coverage effectively prevented Que degradation in solid state, while unmodified LNP@Que (100% GM) exhibited a visible color change from yellow to pink, OA-containing formulations maintained their original coloration across all tested ratios. This protective effect was similarly observed in the solution phase (Figure 5B), confirming that OA modification significantly enhances Que stability against photodegradation in both physical states. Following 6 days of visible light exposure, the remaining Que content (*C*/*C*_0_) (Figure 5C) showed significant variation across formulations: free Que (17.92%), LNP@Que (0% OA, 60.39%), and LNP@Que/OA nanoparticles with 10% (90.37%), 20% (92.11%), and 30% OA (91.78%). These results demonstrate that OA-modified nanoparticles substantially enhanced photochemical stability compared with both free Que and unmodified LNP@Que. The protective effect was particularly notable in formulations containing 10–30% OA, which maintained over 90% of the initial Que content. This improved light stability suggests that LNP@Que/OA nanoparticles could effectively protect Que during manufacturing, storage, and transportation, thereby expanding its potential applications in the food industry.

**Figure 5:**
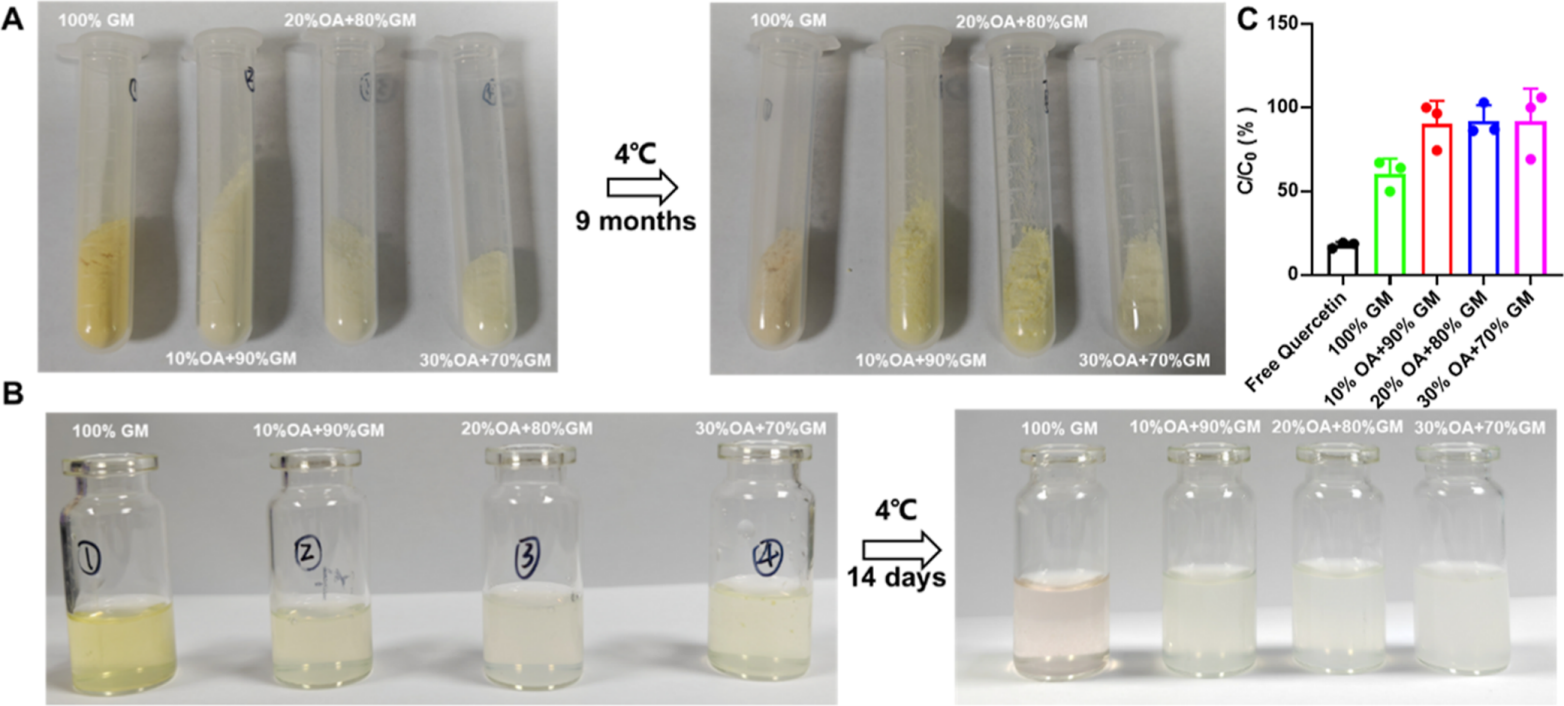
Antidegradation activity of LNP@Que/OA NPs (A) in the solid form and (B) in solution, (C) the antidegradation rate of LNP@Que/OA NPs in solution measured by UV–vis, *n* = 3.

#### 3.4.2 Antioxidant Activity

The antioxidant activity of LNP@Que/OA nanoparticles was assessed through measurement of reducing power using the potassium ferricyanide reduction method, based on the established correlation between reducing capacity and antioxidant potential.^20^ The antioxidant activity assessment (Figure 6) revealed that LNP@Que nanoparticles exhibited the lowest *A*/*A*_0_ values, while OA-modified LNP@Que/OA nanoparticles showed significantly higher values. Notably, both 10% and 30% OA formulations demonstrated similar *A*/*A*_0_ levels, providing direct evidence that OA surface modification effectively enhances the system’s antioxidant capacity.

**Figure 6:**
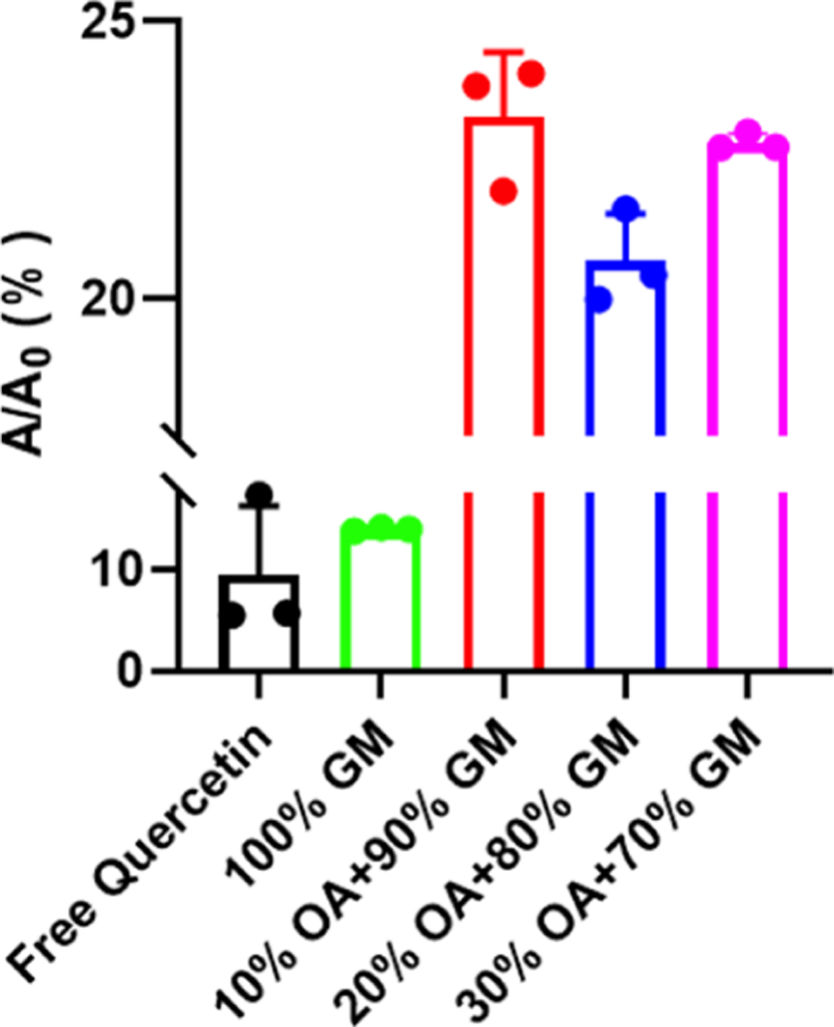
Antioxidant activity of LNP@Que/OA NPs. *n* = 3.

### 3.5 In Vitro and Vivo Safety Assessment

The in vitro hemolytic test was conducted to learn the biocompatibility of nanoparticles; as shown in Figure 7, LNP@Que/OA displayed neglectable hemolysis even at the highest concentration of 0.6 mg/mL, all the hemolysis rate is less than 5%, and it can be considered that the material or substance has good compatibility with blood and will not cause significant hemolysis reactions. Our in vivo safety assessment demonstrated that LNP@Que/OA nanoparticles showed excellent biocompatibility in mouse models. All animals maintained normal activity levels, feeding patterns, and physical appearance throughout the study, with no observed mortality, gastrointestinal symptoms, or behavioral abnormalities. These results preliminarily confirm the nontoxic nature of the nanoparticle formulation.

**Figure 7:**
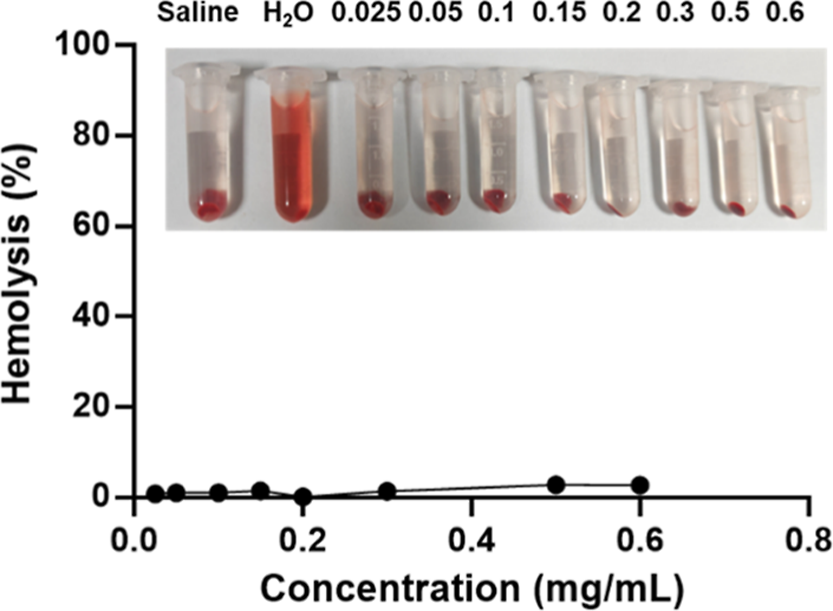
In vitro hemolytic test of LNP@Que/OA.

We further evaluated the systemic toxicity through comprehensive blood biochemistry analysis across all experimental groups. As demonstrated in Figure 8, the serum biomarkers (ALT, AST, urea, and crea) in both quercetin-only and LNP@Que/OA treatment groups showed no statistically significant differences compared to the control group. These hematological findings provide additional evidence supporting the excellent biocompatibility profile of our LNP@Que/OA nanoparticle formulation.

**Figure 8:**
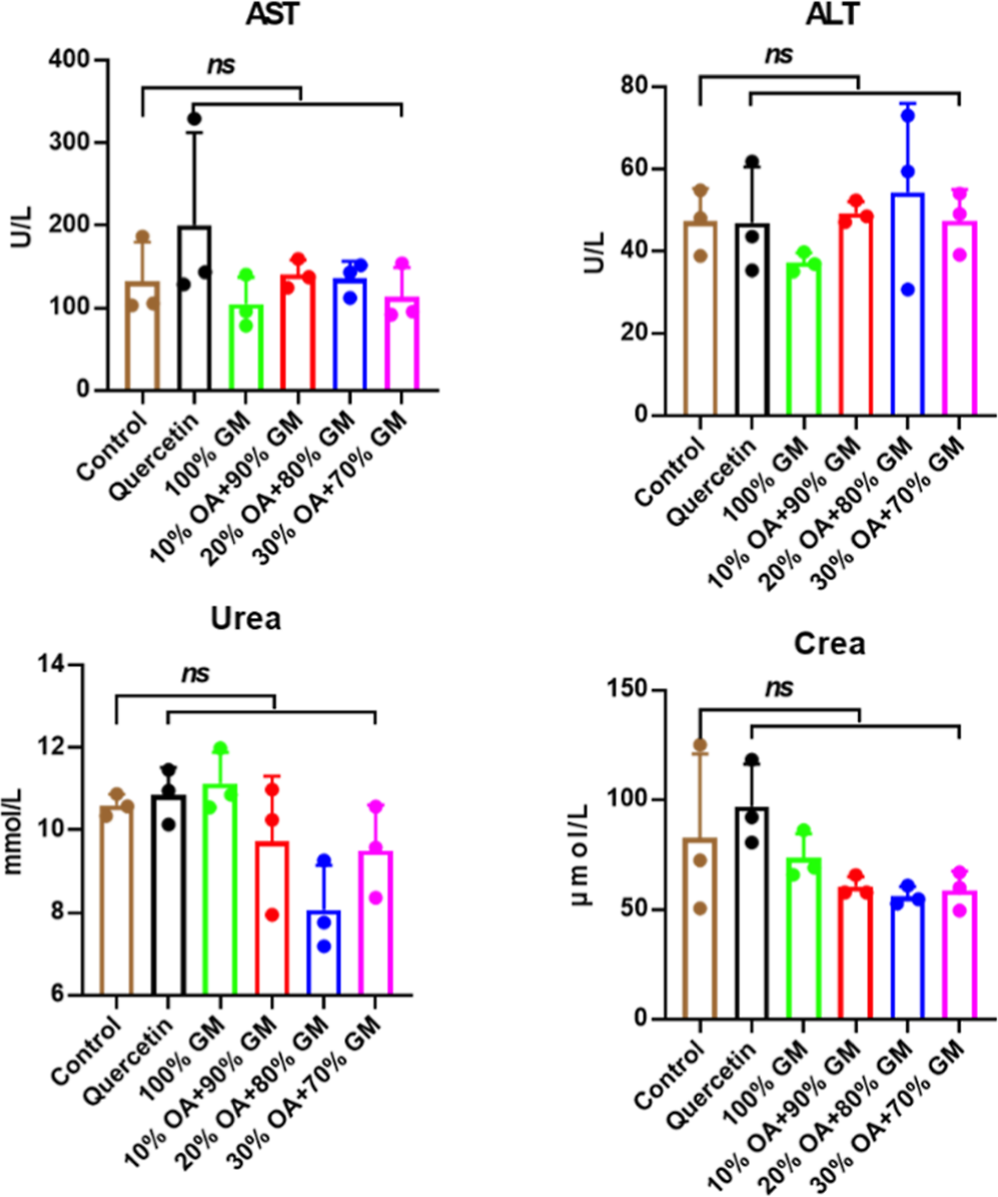
Serum biochemical analysis of different groups, *n* = 3.

Hematoxylin and eosin (H&E) staining, a standard histological technique for evaluating tissue morphology, was employed to assess potential organ toxicity. Following sacrifice, we collected and fixed the major organs (heart, liver, spleen, lungs, and kidneys) in 4% paraformaldehyde for histological processing. The microscopic examination (Figure 9) revealed normal tissue architecture across all organs with no evidence of pathological lesions or structural abnormalities. These histological findings, combined with our previous biochemical and behavioral observations, collectively demonstrate the excellent safety profile of the LNP@Que/OA nanoparticle formulation.

**Figure 9:**
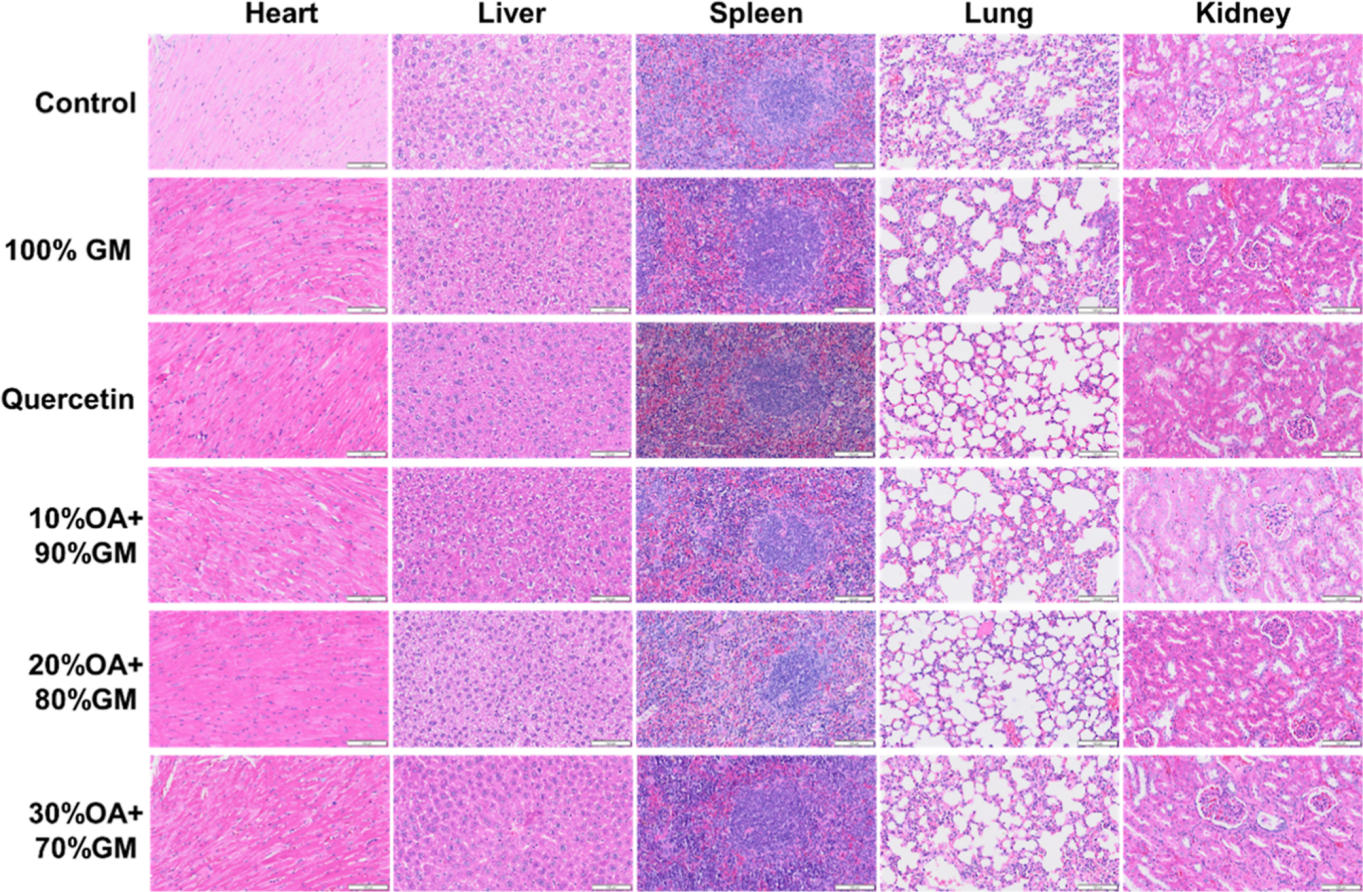
HE results of different groups.

## 4 Conclusion

To overcome Que’s pharmaceutical limitations, we successfully developed a novel solid lipid nanocomplex (LNP@Que/OA) using GM and OA. The optimized GM/OA ratio (9:1) yielded stable nanoparticles with high encapsulation efficiency (98.46%). OA modification significantly enhanced the nanocomplex’s antioxidant capacity, stability, and controlled release properties during simulated digestion. The LNP@Que/OA system developed in this study holds significant promise for diverse pharmaceutical and therapeutic applications, particularly in addressing oxidative stress and inflammation-related disorders. Its enhanced stability, improved bioavailability, and controllable release profile make it particularly suitable for managing neurodegenerative diseases like Alzheimer’s and Parkinson’s through targeted delivery of quercetin’s neuroprotective antioxidants to the brain, as well as for cardiovascular protection in atherosclerosis by reducing LDL oxidation. The formulation shows strong potential for inflammatory conditions including rheumatoid arthritis through localized joint delivery, inflammatory bowel diseases via targeted colonic release, and various dermatological applications by combining quercetin’s anti-inflammatory effects with OA’s wound-healing properties. In preventive medicine, this system could revolutionize nutraceutical formulations by enabling the effective delivery of quercetin in dietary supplements and functional foods targeting aging, immune support, and metabolic syndrome. The biocompatible lipid components and tunable release kinetics allow customization for various administration routes, while OA may provide synergistic benefits in metabolic disorders. To realize this therapeutic potential, future work should focus on disease-specific optimization, comprehensive preclinical validation, GMP-compatible scale-up, and investigation of drug–quercetin interactions. This technology not only addresses current limitations in quercetin delivery but also establishes an adaptable platform for other unstable polyphenolic compounds, offering broad opportunities for advancing treatment strategies across multiple therapeutic areas, where oxidative stress and inflammation play key pathological roles.
